# The widely used *Nicotiana benthamiana* 16c line has an unusual T-DNA integration pattern including a transposon sequence

**DOI:** 10.1371/journal.pone.0171311

**Published:** 2017-02-23

**Authors:** Joshua G. Philips, Fatima Naim, Michał T. Lorenc, Kevin J. Dudley, Roger P. Hellens, Peter M. Waterhouse

**Affiliations:** 1 Centre for Tropical Crops and Biocommodities, Queensland University of Technology, Brisbane, Australia; 2 Institute for Future Environments, Central Analytical Research Facility, Queensland University of Technology, Brisbane, Australia; 3 Institute for Future Environments, Queensland University of Technology, Brisbane, Australia; University of California, Riverside, UNITED STATES

## Abstract

*Nicotiana benthamiana* is employed around the world for many types of research and one transgenic line has been used more extensively than any other. This line, 16c, expresses the *Aequorea victoria* green fluorescent protein (GFP), highly and constitutively, and has been a major resource for visualising the mobility and actions of small RNAs. Insights into the mechanisms studied at a molecular level in *N*. *benthamiana* 16c are likely to be deeper and more accurate with a greater knowledge of the GFP gene integration site. Therefore, using next generation sequencing, genome mapping and local alignment, we identified the location and characteristics of the integrated T-DNA. As suggested from previous molecular hybridisation and inheritance data, the transgenic line contains a single GFP-expressing locus. However, the GFP coding sequence differs from that originally reported. Furthermore, a 3.2 kb portion of a transposon, appears to have co-integrated with the T-DNA. The location of the integration mapped to a region of the genome represented by Nbv0.5scaffold4905 in the www.benthgenome.com assembly, and with less integrity to Niben101Scf03641 in the www.solgenomics.net assembly. The transposon is not endogenous to laboratory strains of *N*. *benthamiana* or *Agrobacterium tumefaciens* strain GV3101 (MP90), which was reportedly used in the generation of line 16c. However, it is present in the popular LBA4404 strain. The integrated transposon sequence includes its 5’ terminal repeat and a transposase gene, and is immediately adjacent to the GFP gene. This unexpected genetic arrangement may contribute to the characteristics that have made the 16c line such a popular research tool and alerts researchers, taking transgenic plants to commercial release, to be aware of this genomic hitchhiker.

## Introduction

Reporter genes have been profoundly important in advancing biological research. In plants, one of the first widely adopted reporter systems utilised beta glucuronidase (GUS) [1], which converts a soluble clear substrate to an insoluble blue precipitate. Unfortunately, this is a destructive histochemical technique. The more recent discovery and application of the green fluorescent protein (GFP) from *Aequorea victoria* allows reporter assays to be live, continuous and non-destructive, and has revolutionised molecular science. GFP was first used in bacteria and animal cells but was rapidly adopted by plant researchers following addition of plant regulatory signals and codon optimisation. The pioneering plant expression constructs, such as *mGFP4*, *mGFP5*, and *mGFP5-ER* [2, 3], have been used extensively in both monocotyledon and dicotyledon plants. For many of these species, different research groups have produced their own GFP-expressing lines and rarely has a single transgenic GFP-expressing line of a species been embraced by a whole research sector. However, the 16c line of *N*. *benthamiana*, from David Baulcombe’s laboratory, is the notable exception. It was generated alongside three other lines (GFP8, GFP17b, and GFPY) [4], which have not been widely distributed, but 16c has been cited in more than 750 publications. These include: exploring virus-plant interactions, RNAi, mobile signals, florigens, grafting, viroids, protein structure and function, protein-protein interactions, human and avian viruses, and silencing suppressors. We have already published and made available the genome and transcriptome sequences of *N*. *benthamiana* [5–7] (www.benthgenome.com), and believe that reporting further details about the location and characteristics of the T-DNA insertion in the 16c line will aid the plant research community.

## Materials and methods

### Plant material

*Nicotiana benthamiana* cultivars LAB [5, 7] and 16c (a generous gift from David Baulcombe, UK) were grown in soil (Plugger custom Mix, Debco, supplemented with Osmocote^®^ slow release fertiliser) in a controlled growth chamber, at constant temperature of 22.5°C, 16 hours day length, 300 μm/m^2^ light and 60% relative humidity.

### DNA extractions

DNA was extracted from approximately 100 mg of freshly ground leaf material using the NucleoSpin^®^ Plant II (Macherey-Nagel) extraction kit as per manufacturer’s instructions with the following modification: 1% v/v β-mercaptoethanol (Sigma-Aldrich) was added to the lysis buffer to inhibit oxidative damage. Extracted DNA was quantified spectrophotometrically using the Nanodrop 2000 (Thermo-Fisher) and an aliquot of 300 ng run on a 1% TAE (tris-acetate-EDTA) agarose gel and visualised under blue light to determine its integrity.

### Genome sequencing

The 16c DNA library was prepared using the TruSeq^®^ Nano Kit (Illumina) as per manufacturer’s instructions and processed on one lane of the NextSeq^®^ 500 (Illumina) housed at the Queensland University of Technology, Central Analytical Research Facility (QUT CARF). Raw reads were filtered prior to alignment; Trimmomatic [8] was used to only keep reads of 150 nt, FastUniq was used to remove PCR duplicates and BBsplit from the BBMap package (https://sourceforge.net/projects/bbmap/) was used to remove reads aligning to mitochondria and chloroplast genomes (*Nicotiana tabacum* GenBank ID BA000042.1 and Z00044.2, respectively). 212,397,922 paired end reads were aligned to pBIN19-mGFP5-ER sequence using Bowtie2 on Galaxy Queensland platform (http://galaxy-qld.genome.edu.au/galaxy).

### RNA extraction and RNAseq

Total RNA was isolated from two week old 16c seedlings using PureLink^®^ RNA Mini Kit as per manufacturer's protocol with a column DNAse treatment. RNA integrity was checked on a 1% TAE gel prior to library preparation. NGS (next generation sequencing) library was prepared using Illumina TruSeq^®^ Stranded Total RNA with Ribo-Zero kit as per manufacturer’s protocol and processed on HiSeq^®^ (Illumina) housed at the Australian Genome Research Facility (AGRF).

### Sanger sequencing of T-DNA insert in 16c

Primers pairs B+E and D+F (Table 1) were used to amplify the genomic DNA sequences flanking the T-DNA LB and RB. PCR amplifications were carried out as follows; an initial denaturation step at 95°C for 3 min, followed by 35 cycles comprising of 95°C for 15 s, 55°C for 15 s, and 72°C for 4 min, followed by a final extension for 6 min at 72°C. Reactions contained 2G Robust HotStart ReadyMix (KAPA), 0.5 μM each of forward and reverse primer, 10 ng of 16c DNA as template and volume made up to 20 μL with molecular grade water. The resulting PCR amplicons were gel excised and cloned into pGEM^®^-T Easy (Promega) using standard molecular techniques. Four clones were sequenced using Sanger sequencing.

**Table 1:** List of primers used in this study.

Primer ID	Primer sequence	Description
A	AGGAATATATGTTGGGTTTGAATC	*N*. *benthamiana* flanking RB in 16c Fwd2
B	AATTCTGGAAATATCAAAGGTG	*N*. *benthamiana* flanking RB in 16c Fwd1
C	GCTTAGCTCATTAAACTCCAGA	NOS promoter Rev
D	CTCGGCCACAAGTTGGAATA	Internal primer for *mGFP5-ER* Fwd
E	GGTCTTGAAGTTGGCTTTGATG	Internal primer for *mGFP5-ER* Rev
F	CACTTGTGAGGGGAGAATATAA	*N*. *benthamiana* flanking LB in 16c Rev

To ascertain to which scaffold the T-DNA insertion had integrated into, primer pair A+C (Table 1) were used to generate a 1.7 kb amplicon which was analysed by Sanger sequencing as previously described.

## Results

### Genomic location of the T-DNA

**Fig 1:**
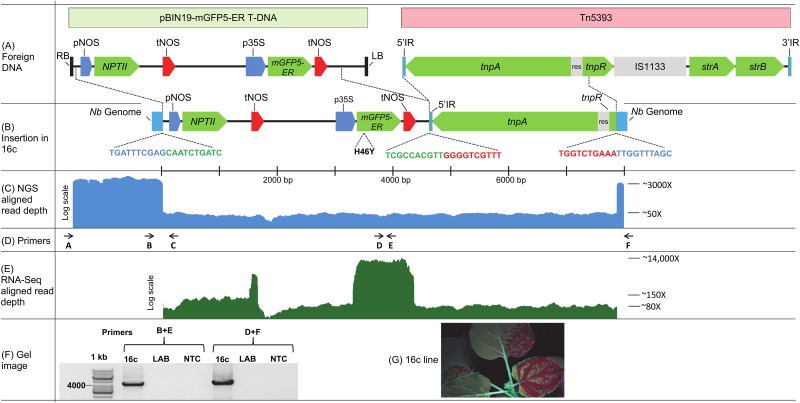
Schematic of pBIN19-mGFP-ER and Tn5393 and their integration site in 16c. **(A–B)** Schematic of genes within the T-DNA and transposon integrated in 16c genome. The sequence junctions between *N*. *benthamiana* genome-T-DNA-transposon-*N*. *benthamiana* genome are highlighted, GenBank accession number KY464890. **(C)** Log scale density plot of sequence reads aligning to T-DNA, transposon and flanking *N*. *benthamiana* genomic regions. **(D)** Location of primers used for amplification of various regions to confirm site of integration. **(E)** Log scale density plot of RNAseq reads aligning to T-DNA and transposon. **(F)** Gel image showing the endpoint (35 cycles) PCR amplicons of the T-DNA insert in 16c with LAB *N*. *benthamiana* used as a control. Expected sizes with primer pair B+E is 4174 nt and D+F is 4167 nt. NTC, no template control, 1 kb DNA Ladder (GeneRuler^™^). **(G)** Leaves of *N*. *benthamiana* 16c photographed under UV light, showing mobile silencing, 14 days after local induction of RNAi at a lower leaf. Abbreviations are: RB: Right Border, pNOS: nopaline synthase promoter, *NPTII*: neomycin phosphotransferase II encoding gene, tNOS: nopaline synthase terminator, p35S: Cauliflower mosaic virus 35S promoter. IR: inverted terminal repeat, *tnpA* transposase gene, res: recombination region, *tnpR* resolvase gene, IS1133: an insertion element, *strA* and *strB*: streptomycin resistance genes, Nb Genome: Flanking DNA of *N*. *benthamiana*.

Genomic DNA was extracted from young leaves of the 16c line and used to generate a genomic DNA library. This was sequenced on an Illumina NextSeq^®^ 500 machine and, after trimming and filtering, produced 212,397,922 paired end reads. The reads were aligned to the sequences of both pBin-35S-mGFP5 and pBin-35S-mGFP5-ER. This revealed that, in contrast to the original report [4], the GFP gene within the T-DNA encodes a protein possessing both an ER targeting signal peptide and an ER retention signal. These signal-encoding sequences and all but one nucleotide (C->T, H46Y) in the body of the GFP gene are identical to those of *mGFP5-ER* [3]. Many of the reads aligned perfectly with the transfer region of the Ti plasmid and formed one contig but none aligned with left border (LB) or right border (RB) sequences (Fig 1). Furthermore, neither terminus of the contig had T-DNA-like sequences. At the potential RB and LB ends are 115 nt and 108 nt of non-T-DNA sequence, respectively. Using BLAST to compare the 115 nt sequence with the genome assembly of the LAB strain (www.benthgenome.com), produced many alignments but gave only one 100% uninterrupted match. This was with a region of Nbv0.5scaffold4905. The 108 nt sequence gave no significant alignments from a BLAST search of the *N*. *benthamiana* genome but produced several good alignments with bacterial sequences in the NCBI non-redundant database (https://blast.ncbi.nlm.nih.gov/Blast.cgi). To investigate this further, two sets of primer pairs were used to amplify regions of the 16c genome (Fig 1D). One set used a primer binding within the 115 nt sequence (primer B) and the other within the *mGFP5-ER* gene (primer E). The second primer set utilised one (primer F) designed to bind to a region 175 nt downstream of primer B site on Nbv0.5scaffold4905 and the other (primer D) binding within the GFP gene. Primers B+E produced an amplicon of 4.17 kb, and primers D+F gave a 4.17 kb product (Fig 1F). These were cloned and sequenced. The B+E amplicon sequence matched the expected region of the contig sequence but the D+F product was much larger than anticipated and contained 3.2 kb of sequence matching a bacterial transposon (Fig 1B).

Although the 115 nt sequence matches perfectly with Nbv0.5scaffold4905, it also has near-perfect matches with >100 different scaffolds in both the benthgenome.com and solgenomics.net assemblies. Therefore, a primer (primer A) binding ~1.5 kb upstream of the primer B site on Nbv0.5scaffold4905 was synthesised and used in conjunction with a primer binding to the NOS promoter (primer C) to test for and amplify a longer genomic region (Fig 1D). The sequence of the 5’ 1.5 kb of the 1.7 kb amplicon matched perfectly with Nbv0.5scaffold4905 but poorly with all other scaffolds. This unequivocally identifies Nbv0.5scaffold4905 as representative of the region of the 16c genome harbouring the mGFP5-ER T-DNA insert. The results also show that 58 nt has been lost from the plant’s genome during T-DNA integration.

**Fig 2:**
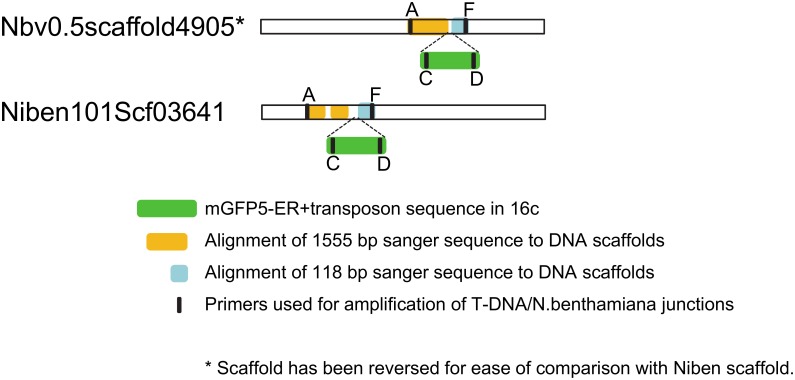
Schematic showing the alignment of Sanger sequences to scaffolds in benthgenome.com and solgenomics.net.

Interrogating the solgenomics.net assembly using BLAST with this 1.5 kb sequence produced multiple hits on the Niben101Scf03641 scaffold. Pairwise comparison of the sequences produced two regions of almost perfect alignment (1008 nt and 523 nt), separated by a 41 nt region that appears to be due to an insertion in Niben101Scf03641 (Fig 2). The region of *N*. *benthamiana* sequence surrounding the T-DNA insertion site (115+57+108 nt) matches many scaffolds and sometimes many times within a scaffold. This suggests that the sequence is distributed widely throughout the genome. Its repeated presence is also evident from the great apparent depth of coverage for the sequences adjacent to the T-DNA insertion (Fig 1C). If they were only present at this location, their depth of coverage should be similar to that for the T-DNA (i.e. ~50x). However, their apparent depth is approximately 60 times greater (~3000x).

To investigate the expression of genes inserted at this locus, an RNAseq library was prepared from 16c seedlings. The resulting NGS library contained 40,940,956 100 bp single end reads which were aligned to the T-DNA+transposon sequence. This confirmed the high expression level of *mGFP5-ER* compared to the *NPTII* gene (Fig 1E).

### Unusual co-integration of T-DNA with a partial transposon sequence

A *de novo* genome assembly was constructed using the 16c reads. This produced a contig containing the same pBIN19-mGFP5-ER T-DNA sequence that we found by mapping the 16c raw reads onto the plasmid sequence. Assuming that the T-DNA cut sites are between the third and fourth nucleotide of the 25 bp border repeat sequence [9], there is a 56 bp T-DNA truncation at the RB and a 444 bp truncation at the LB. There is also a co-integrated partial transposon sequence (Fig 1B). This sequence appears to be part of the class II Tn3-type transposable element, Tn5393 (GenBank Accession No. M96392.1), and contains its 5' inverted terminal repeat (IR), transposase (*tnpA*) gene, recombination region (res), and 128 nt of its resolvase (*tnpR*) gene. While an intact Tn5393 transposon also harbours two streptomycin resistance genes, an insertion element and a 3' IR [10] (Fig 1A), these components are not integrated into 16c either in Nbv0.5scaffold4905 or elsewhere in the genome, as their sequences are not present in the entire 16c read library. Searching the genome of non-transgenic *N*. *benthamiana* failed to detect any Tn5393 sequences but they could be found in the fire blight bacterium, *Erwinia amylovora* [10] and in plasmids borne by a range of other bacteria [10–12]. This suggests that the transposon sequences adjacent to the T-DNA integration in 16c have been translocated from a bacterial source. While *A*. *tumefaciens* strains GV3101 (MP90) and EHA105 do not possess this transposon, it is present in strain LBA4404 [13].

The original description of the generation of *N*. *benthamiana* line 16c [4] reported a simple 3:1 inheritance of the GFP transgene, from a heterozygous parent, and a single band in genomic blots. These results are indicative of a single T-DNA insertion locus. The absence of *N*. *benthamiana*/T-DNA (+Tn5393) junctions in the 16c library sequence reads, other than the two in Nbv0.5scaffold4905, is consistent with this interpretation. Furthermore, there were no read sequences that suggest a complex integration of more than one copy of the T-DNA at the same locus. The complete sequence of the 16c T-DNA+partialTn5393 locus has been lodged at GenBank with the accession number KY464890 and it is also available on a dedicated page of benthgenome.com.

## Discussion

Over the last decade, *Arabidopsis thaliana* and *Oryza sativa* have been the popular model species for studying molecular mechanisms in plant biology. Both are diploid, have fully sequenced small genomes, are easy to transform and have a broad range of available mutants. The T-DNA insertion lines of *A*. *thaliana*, in particular, have been very useful. *N*. *benthamiana* has been used extensively for transient gene expression in the study of many mechanisms, but much less so using stable transgenesis because of the complexity of its allopolyploid genome, lack of genomic sequence information, and the dearth of well-defined mutants [14]. However, there is now considerable interest in how mechanisms operate in polyploid genomes, as most crop species are polyploids. There are also two independent draft genome assemblies of *N*. *benthamiana* (benthgenome.com & solgenomics.net) from an Australian and American research group, respectively, and CRISPR-Cas technology appears capable of efficiently generating targeted *N*. *benthamiana* mutants [15, 16]. The *GFP* gene in 16c has been used in many studies including those on the induction and maintenance of epigenetic changes [17, 18], and as a reporter for post-transcriptional gene silencing [19]. However, many of these assays have relied on incomplete or inaccurate details of the GFP locus. In order to furnish researchers with more accurate details for future studies utilising 16c, we determined the sequence and location of this locus and discovered some unexpected details. We confirmed, at a sequence level, the assertion that the *GFP* transgene is in a single locus and that it is highly expressed when compared to the *NPTII* selectable marker gene. We determined that the *GFP* transgene has the ER targeting and retention signals of *mGFP5-ER* and not the untargeted *GFP* design of *mGFP5*, as reported in the original description [4]. We also identified a sequence variation that alters an amino acid residue within the GFP protein that is not a reported change in *mGFP5-ER* arising from *mGFP4-ER*.

Interestingly, when mapping the T-DNA to Nbv0.5scaffold4905 and to Niben101Scf03641, we found a 41 nt insertion in the solgenomics.net assembly. This insertion may reflect either a misassembly or a genuine difference between the two isolates. If it is the latter case, this suggests that the background of 16c is more similar to the Australian *N*. *benthamiana* LAB isolate than to the one used by the American research group [20].

The most surprising finding was that a large section of an alien transposon, including its *tnpA* gene, has been inserted adjacent to the T-DNA at its integration site. This transposon, Tn5393, is present in plasmids borne by a range of bacterial species [10–12] including some *A*. *tumefaciens* strains [13, 21]. A recent study [13] of over 300 transgenic rice lines, transformed using the LBA4404 strain of *A*. *tumefaciens*, found this transposon to have co-integrated into the genome of 26% of them. The Tn5393 sequence is often integrated immediately adjacent to the T-DNA and often as an incomplete copy. This almost exactly mirrors the situation in 16c. However, the *A*. *tumefaciens* strain reported [4] to have been used to create 16c was GV3101 (MP90), which does not harbour the transposon sequence [13]. Perhaps, LBA4404 or some other Tn5359-bearing *A*. *tumefaciens* strain was mistakenly used, or was contaminating the GV3101 (MP90) culture used, for the transformation.

The 16c line of *N*. *benthamiana* has been employed widely to track the systemic mobility of gene silencing, after local induction (Fig 1G), and to assay suppression of gene silencing by potential viral suppressor proteins [22, 23]. Moreover, plant transposons are often the target of transcriptional and post-transcriptional gene silencing [24, 25]. This raises the possibility that the bacterial transposon sequence adjacent to the GFP gene in 16c has an enhancing effect on the plant’s silencing response, once it is triggered. This could explain why 16c is such a sensitive epigenetic research tool. Until now researchers have been unaware of 16c’s integrated bacterial transposon sequence. We envisage that future studies using 16c will not only monitor the GFP gene but also examine the chromatin status of the adjacent transposon sequence. Indeed, if the sequence is found to enhance the induced silencing reporter system, it could become a blueprint for the design of similar reporter systems in other species. Furthermore, with the current regulatory climate requiring clean, well-documented transgenic insertions in commercialized GMO crops, we suggest that it would be prudent to assay for this transposon to ensure that it has not been unwittingly introduced into transgenic lines destined for commercial release.
